# The long non‐coding RNA PCAL7 promotes prostate cancer by strengthening androgen receptor signaling

**DOI:** 10.1002/jcla.23645

**Published:** 2020-11-21

**Authors:** Zhihui Li, Jingfei Teng, Zhuomin Jia, Guohui Zhang, Xing Ai

**Affiliations:** ^1^ Department of Urology The Seventh Medical Center of PLA General Hospital Beijing China

**Keywords:** HIP1, LncRNA, PCAL7, prostate cancer

## Abstract

**Background:**

The prostate cancer (PCa) has been a global problem to men health. Notably, the androgen receptor (AR) is essential for both normal development of prostate and prostate cancer progression.

**Methods:**

The RNA sequencing was used to identify the novel long non‐coding RNA (lncRNA) termed PCAL7. The RT‐qPCR was performed to quantify PCAL7 expression. Migration and proliferation assays were used to examine the function of PCAL7. Fluorescence in situ hybridization (FISH) was used to determine subcellular localization.

**Results:**

By RNA sequencing, the differentially expressed lncRNAs were identified (top 10 upregulated lncRNAs: PCAL7, AC083843.1, CTC‐338M12.3, RP11‐443B7.1, RP11‐1008C21.2, RN7SL329P, RP4‐773N10.4, RP11‐264B17.2, KB‐1507C5.2, and RP11‐20B24.6; top 10 downregulated lncRNAs: RP11‐77H9.2, RAB11FIP1P1, AP001625.6, CTA‐217C2.1, RP11‐603J24.7, RP11‐315I20.1, AC092839.1, RP4‐758J18.10, RP11‐259O2.3, and HMGN2P17). PCAL7 was the lncRNA with the highest fold upregulation and significantly correlated with AR signaling during prostate cancer progression. The AR‐regulated PCAL7 was abundantly overexpressed in prostate cancer tissues and AR‐dependent cell lines. PCAL7 knockdown inhibited cell migration and proliferation. Consistently, the migration and proliferation were promoted by PCAL7 overexpression. PCAL7 depletion via antisense oligonucleotides (ASOs) markedly suppressed AR signaling and tumor growth. Mechanistically, PCAL7 interacted with Huntingtin‐interacting protein 1 (HIP1) to stabilize HIP1. Therefore, PCAL7 could advance AR signaling via a novel positive feedback loop.

**Conclusion:**

Our experiments support an oncogenic role for PCAL7 which promotes prostate cancer progression suggesting PCAL7 may serve as a potential therapeutic target.

## Graphical abstract

The novel lncRNA PCAL7 can promote prostate cancer progression via stabilizing HIP1. The androgen receptor (AR) can induce PCAL7 expression. Since HIP1 decreases androgen receptor degradation, the AR‐PCAL7‐HIP1 axis creates a novel positive feedback loop to advance prostate cancer development.

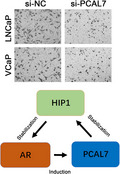

## 1 INTRODUCTION

The prostate cancer (PCa) is classified as a non‐cutaneous type of cancer which affects men health worldwide.^1^ The transcription factor androgen receptor (AR) is a crucial modulator for oncogenesis.^2^ Androgen receptor regulates the expression of multiple genes and is highly effective for metastatic prostate cancer treatment.^3^ Most primary prostate cancer cases show AR dependence and are responsive to androgen‐deprivation therapy.^4^ Even for castration resistance prostate cancer (CRPC) cases, prostate cancer progression is also substantially dependent on AR signaling pathway.^5^ Therefore, understanding AR‐mediated tumorigenesis during prostate cancer progression is highly important for prostate cancer therapy.

The majority of human genome is pervaded with non‐protein coding sequences, and some transcripts are classified into long non‐coding RNAs (lncRNAs) with more than 200 bp in length.^6^ Recent studies have uncovered many lncRNAs with specific roles for prostate cancer development.^7^, ^8^, ^9^ For example, the steroid receptor RNA activator (SRA) is regarded as an AR regulator and potentiates prostate cancer progression.^10^ The AR‐induced novel lncRNA named LINC00844 inhibits prostate cancer metastasis by increasing AR chromosome binding to induce NDRG1, a well‐known metastasis suppressor.^8^ The AR‐regulated lncRNA nuclear enriched abundant transcript 1 (NEAT1) can be recruited to the chromatin and epigenetically promotes the expression of prostate cancer‐associated genes.^9^ In addition, AR‐induced ARLNC1 associates with androgen receptor mRNAs and stabilizes AR transcripts to augment AR signaling.^7^ Although current knowledge about lncRNAs in prostate cancer is expanding, numerous lncRNAs remain unidentified with respect to AR‐mediated regulation.

By lncRNA sequencing, we explored a novel lncRNA related to prostate cancer progression named as prostate cancer‐associated lncRNA on chromosome 7 (PCAL7). We found that PCAL7 expression is increased in prostate cancer tissues and primarily distributed in cytoplasm. PCAL7 overexpression promoted migration and proliferation of prostate cancer cells. AR directly transcriptionally induces PCAL7 expression. PCAL7 can interact with HIP1 via RNA immunoprecipitation followed by mass spectrometry (MS). The PCAL7‐HIP1 association stabilizes HIP1 proteins to augment AR signaling. We designed several antisense oligonucleotides (ASOs) to reduce PCAL7 expression and ASOs targeting PCAL7 strongly impairs in vivo tumor growth. Taken together, we identified a novel AR‐dependent lncRNA PCAL7 and PCAL7 may serve as a potentially therapeutic target against prostate cancer development.

## 2 MATERIALS AND METHODS

### 2.1 Cell lines

All cell lines were obtained from Shanghai Institute of Cell Biology and maintained in DMEM (Sigma) with 8% fetal bovine serum (FBS) and 100 μg/mL streptomycin. All cells were mycoplasma negative. Cells in androgen stimulation experiments were first staved with charcoal‐deprived serum for 36 hours and stimulated with 10 nmol/L dihydrotestosterone (DHT) as indicated.

### 2.2 Human samples

The human prostate cancer and normal adjacent tissue samples were collected from January 2017 to June 2019 by prostatectomy (N = 104). Samples were stored at −80°C immediately after resection before use. Written informed consent forms were obtained from all patients for academic use. Experiments related to human samples were formally approved by Institutional Review Board at the Seventh Medical Center of PLA General Hospital and conform to Declaration of Helsinki.

### 2.3 Reverse transcription quantitative‐PCR (RT‐qPCR)

Extracted RNAs were obtained using the TRIzol kit (Invitrogen). The PrimeScript RT Reagent Kit with gDNA Eraser (Takara) was utilized for synthesizing the first strand cDNA. All primers used in current work were designed and purchased from Takara (Table S1). ABI 3000 system was used for quantifying RNA.

### 2.4 Statistics

Statistical analyses were performed using SPSS (version 16, SPSS, Inc). Data were represented by mean ± SD. Statistical significance was determined by Mann‐Whitney (two groups) or Kruskal‐Wallis rank sum test (multiple groups) as specified. *P* < .05 was considered significant.

## 3 RESULTS

### 3.1 PCAL7 as a novel lncRNA in prostate cancer

**FIGURE 1:**
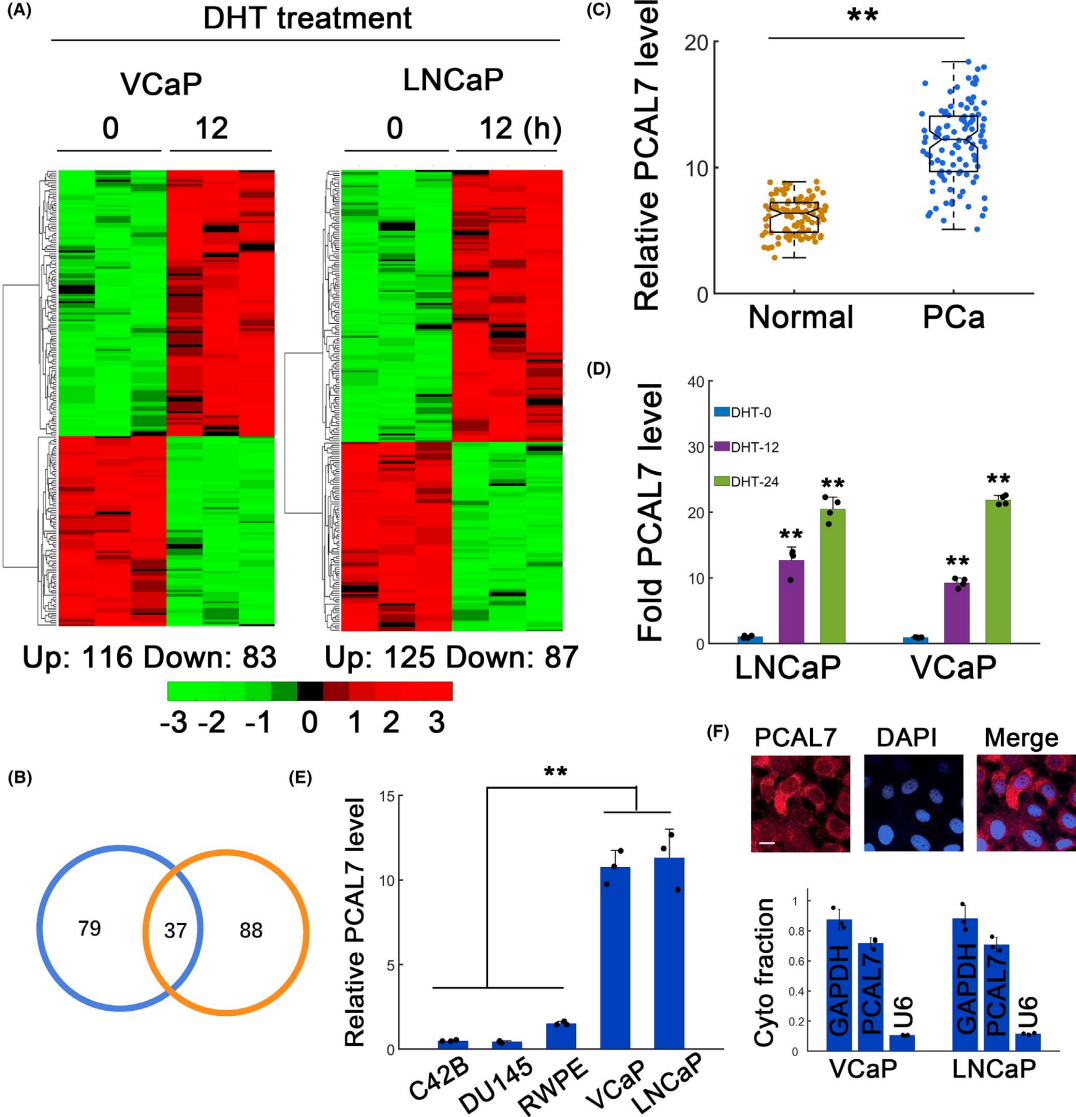
Identification of lncRNA PCAL7. A, RNA‐seq data for androgen regulated transcriptome in LNCaP and VCaP cells after 10 nmol/L dihydrotestosterone (DHT) treatment. The heatmaps depicted differentially expressed lncRNAs in VCaP cells (left) and LNCaP cells (right). B, Venn plot to identify overlapped upregulated genes from both RNA‐seq datasets. C, PCAL7 expression in normal adjacent tissues and PCa tissues. n = 104. D, Temporal PCAL7 expression after DHT treatment for LNCaP and VCaP cells. The black dots represented individual measurements. E, Relative PCAL7 transcript expression in different prostate cancer cell lines. F, Fluorescence in situ hybridization (FISH) for PCAL7 localization in LNCaP cells (top). Scale bar: 50 μm. Cytoplasmic fractionation of PCAL7 transcripts in LNCaP and VCaP cells. *GAPDH* and *U6* RNAs were used as cytoplasmic or nuclear control, respectively. **P < .01

To identify novel AR‐regulated lncRNAs, we performed RNA sequencing (RNA‐seq) on AR‐dependent VCaP and LNCaP cells. VCaP and LNCaP cells were stimulated with AR ligand (10 nmol/L DHT) for 12 hours (Figure 1A). 5208 lncRNAs were screened, and consistently regulated lncRNAs were identified (Figure 1A). Overlapping study showed that 37 lncRNAs were consistently upregulated in LNCaP and VCaP cells (Figure 1B). Since ENSG00000232533 exhibited the highest fold expression, we chose it for further assays. We termed it as prostate cancer‐associated lncRNA on chromosome 7 (PCAL7). PCAL7 was located on chromosome 7:143,379,692‐143,380,495 reverse strand with one annotated transcript (www.ensembl.org). The transcript (ENST00000429630.1) was 468 bp in length. Evaluating the coding potential suggested that it had low coding probability (0.0321009, via http://lilab.research.bcm.edu/cpat/index.php). PCAL7 was also significantly elevated in prostate cancer compared with normal adjacent tissues (Figure 1C). Indeed, DHT treatment could faithfully induce PCAL7 expression at both 12 and 24 hours in LNCaP and VCaP cells (Figure 1D). PCAL7 was not significantly correlated with age but significantly correlated with tumor size, metastasis, and TNM stages (Table S2). We identified that PCAL7 was highly expressed in AR‐dependent prostate cancer cell lines compared to AR‐less or ‐negative cells (Figure 1E). PCAL7 fluorescence signals were primarily located in cytoplasm (Figure 1F, top). Subcellular distribution showed that most PCAL7 was indeed distributed in cytoplasm (Figure 1F, bottom). These data suggested that the novel lncRNA PCAL7 was highly accumulated in prostate cancer with dominant distribution in cytoplasm.

### 3.2 AR induces the expression of PCAL7

**FIGURE 2:**
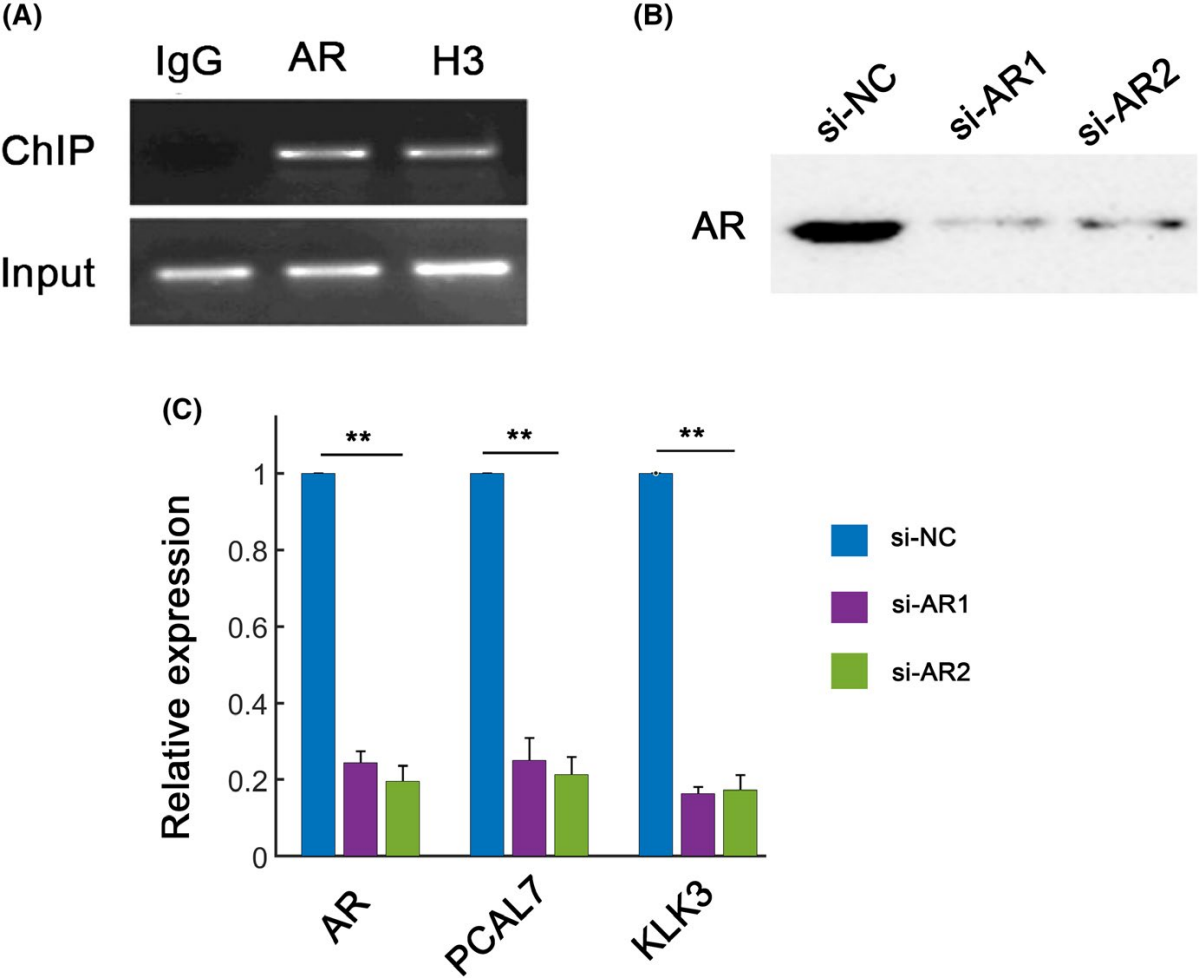
PCAL7 is regulated by AR. A, ChIP assays for AR. The results showed that AR bound to the upstream region of PCAL7. IgG serves as a negative control, whereas H3 serves as a positive control. B, RNA silencing efficiency for AR using siRNA negative control (si‐NC) and two specific siRNAs targeting AR (si‐AR1 and si‐AR2). C, Expression of AR and its target gene (*KLK3*) in LNCaP cells transfected with si‐NC or two siRNAs for AR. n = 3 independent experiments. **P < .01 by ANOVA with Duncan *post hoc* comparisons

Since PCAL7 was highly expressed in AR‐dependent prostate cancer cells, we then inspected whether the promoter region of PCAL7 can be occupied by AR. The ChIP results revealed that AR can directly bind to the promoter region of PCAL7 (Figure 2A). We then evaluated the effect of AR silence on PCAL7 expression using two si‐*AR* oligos (si‐*AR*1 and si‐*AR*2, Figure 2B). Both siRNAs significantly decreased AR expression (Figure 2B). Androgen receptor silence dramatically decreased the expression of *AR* and PCAL7, along with a canonical AR target gene *KLK3* ^11^ (Figure 2C). These results demonstrated that AR can induce PCAL7 expression.

### 3.3 PCAL7 interacts with and stabilizes HIP1

**FIGURE 3:**
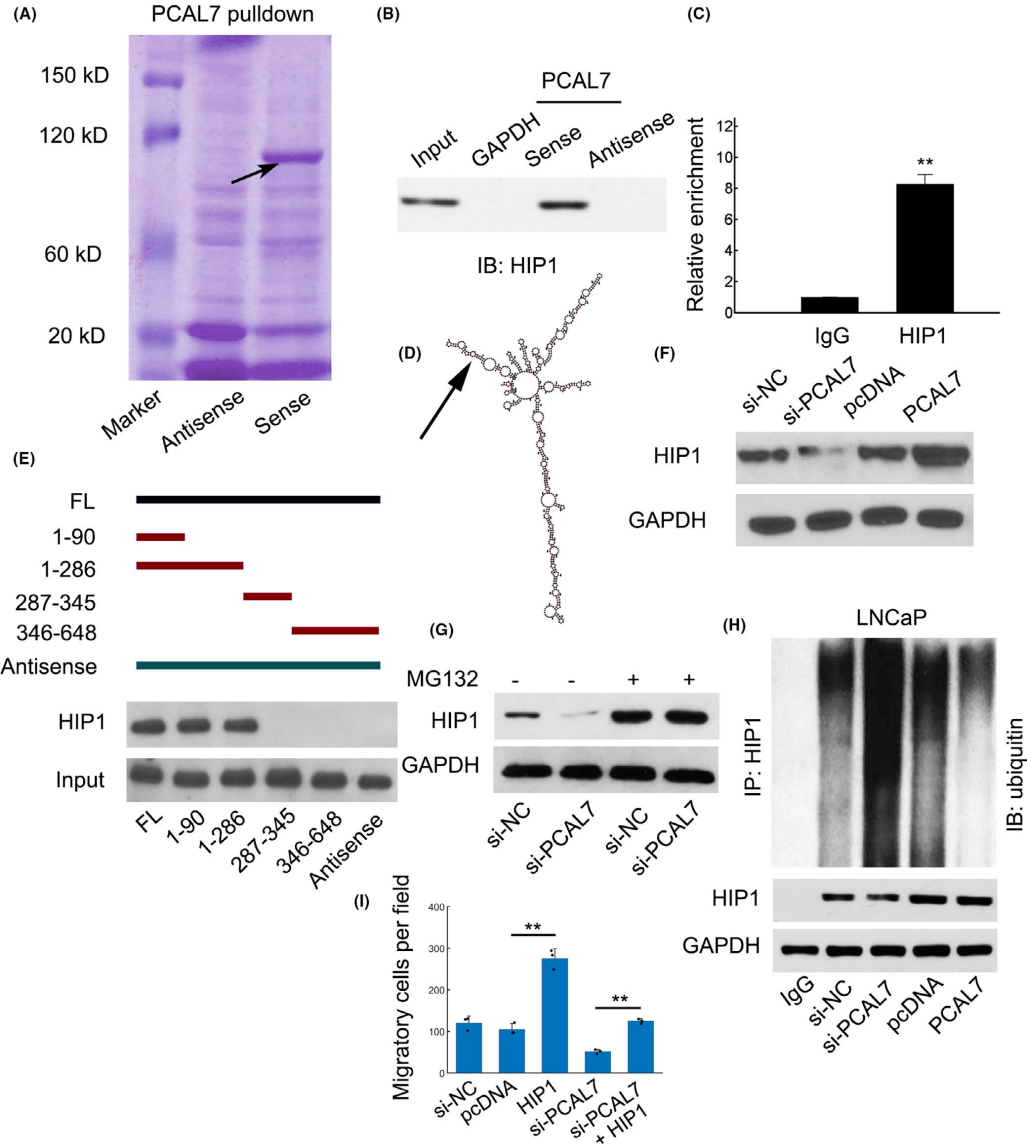
PCAL7 interacted with HIP1. A, Biotin labeled PCAL7*‐*sense and antisense probes incubated in LNCaP lysates. The indicated band (black arrow) was excised and subject to mass spectrometry (MS) verification. B, Immunoblots to identify the interaction between PCAL7 and HIP1. C, RIP assays were performed with antibody against HIP1 followed by qPCR. D, Predicted secondary structure of lncRNA PCAL7 using the online tool http://rna.tbi.univie.ac.at//cgi-bin/RNAWebSuite/RNAfold.cgi. E, Different truncated PCAL7 constructs to identify the domain for HIP1 interaction. F, Western blot assay for HIP1 protein levels by PCAL7 silence or overexpression. G, LNCaP cells were transfected with si‐NC or si‐PCAL7 with or without MG132 (20 μmol/L) treatment followed by immunoblots for HIP1. H, Cell lysates from LNCaP cells with si‐NC, si‐PCAL7, empty pcDNA3.1 vector (pcDNA), or pcDNA‐PCAL7 (PCAL7) were first immunoprecipitated with HIP1 followed by immunoblots against ubiquitin or HIP1. I, Migration assay in LNCaP cells transfected with indicated siRNAs or pcDNA vectors. **P < .01

Notably, the lncRNAs can exert their functions by RNA‐protein interactions.^12^ To unravel potential target for PCAL7, RNA pull‐down followed by mass spectrometry (MS) was performed. The band approaching 120 kDa was resected and analyzed (Figure 3A). A total of six putative proteins were identified, and Huntingtin‐interacting protein 1 (HIP1) was confirmed as the target for PCAL7 binding by immunoblots (Figure 3B and Table S3). The interaction between PCAL7 and HIP1 was also verified using RIP assay (Figure 3C). Various PCAL7 truncated mutants were generated to evaluate the potential binding region based on the predicted secondary structure (Figure 3D, http://rna.tbi.univie.ac.at//cgi-bin/RNAWebSuite/RNAfold.cgi). Results showed that the N‐terminal arm may contribute to PCAL7 and HIP1 binding (Figure 3D,E, the arm was indicated by an arrow). We altered PCAL7 expression using siRNAs or pcDNA vectors (Figure S1A,B). We found that PCAL7 silence or overexpression did not affect the *HIP1 mRNA* levels (data not shown). Instead, PCAL7 levels positively regulated HIP1 protein abundance (Figure 3F). Furthermore, HIP1 degradation was inhibited after treatment with a proteasome inhibitor MG132 suggesting that PCAL7 inhibited proteasome‐mediated HIP1 degradation (Figure 3G). Ubiquitination of HIP1 was strongly inhibited when PCAL7 was overexpressed whereas PCAL7 knockdown dramatically increased ubiquitin ligation of HIP1 (Figure 3H). HIP1 overexpression increased cellular migration, whereas PCAL7 silence decreased the migratory capacity (Figure 3I). As expected, HIP1 overexpression can partially counteract the effect of PCAL7 silence for prostate cancer cell migration (Figure 3I). Previous work has demonstrated that the HIP1 protein associates with AR, reduces AR degradation, and elevates AR transcriptional activity.^13^ Consistently, PCAL7 depletion strongly decreased AR expression (Figure S1C) and AR reporter gene activities (Figure S1D). These results suggested that PCAL7 can interact with and stabilize HIP1 protein to promote AR signaling.

### 3.4 Evaluation of in vitro effect of PCAL7

**FIGURE 4:**
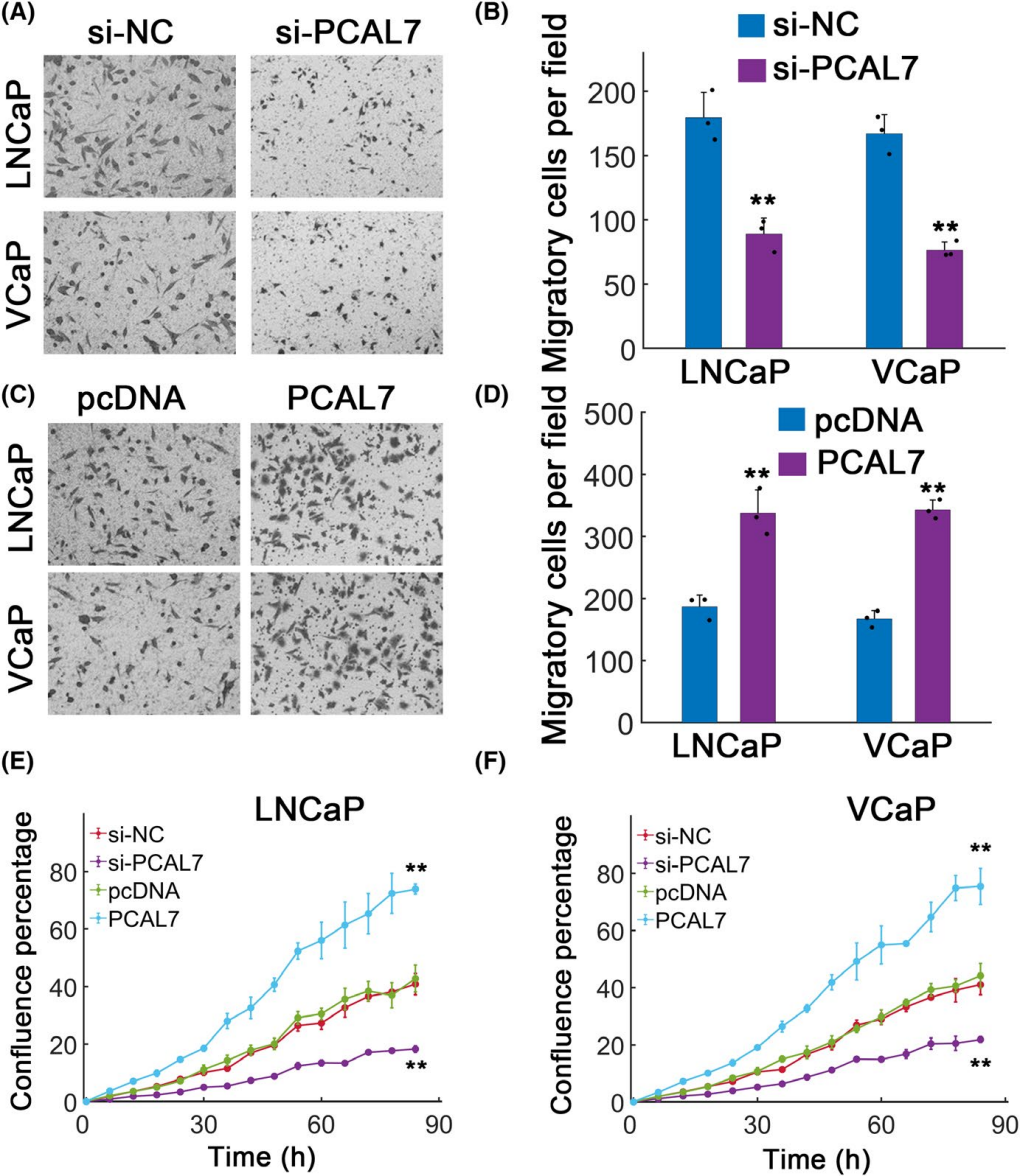
In vitro effect of PCAL7. A, Migration assay for DHT (10 nmol/L)‐treated LNCaP and VCaP cells with or without PCAL7 knockdown. B, Quantification of results in (A). C, Migration assay for DHT‐treated LNCaP and VCaP cells with or without pcDNA mediated PCAL7 overexpression. pcDNA: empty pcDNA vector; PCAL7: pcDNA‐PCAL7. D, Quantification for (C). E, Cell proliferation assay for LNCaP cells transfected with si‐NC, si‐PCAL7, pcDNA3.1 (pcDNA), or pcDNA‐PCAL7 (PCAL7). F, Similar as in (E) in VCaP cells. **P < .01. The black dots indicated measurements

We then investigated the in vitro role of PCAL7 in prostate cancer cells. PCAL7 silence markedly inhibited migration in LNCaP and VCaP cells (Figure 4A,B, *P* < .01). As expected, ectopic PCAL7 overexpression significantly facilitated LNCaP and VCaP cell migration (Figure 4C,D, *P* < .01). PCAL7 knockdown also substantially decreased LNCaP and VCaP cell proliferation, whereas PCAL7 overexpression advanced cell proliferation (Figure 4E,F). These results showed that PCAL7 promoted migration and proliferation of prostate cancer cells in vitro.

### 3.5 PCAL7 knockdown inhibits tumor growth

**FIGURE 5:**
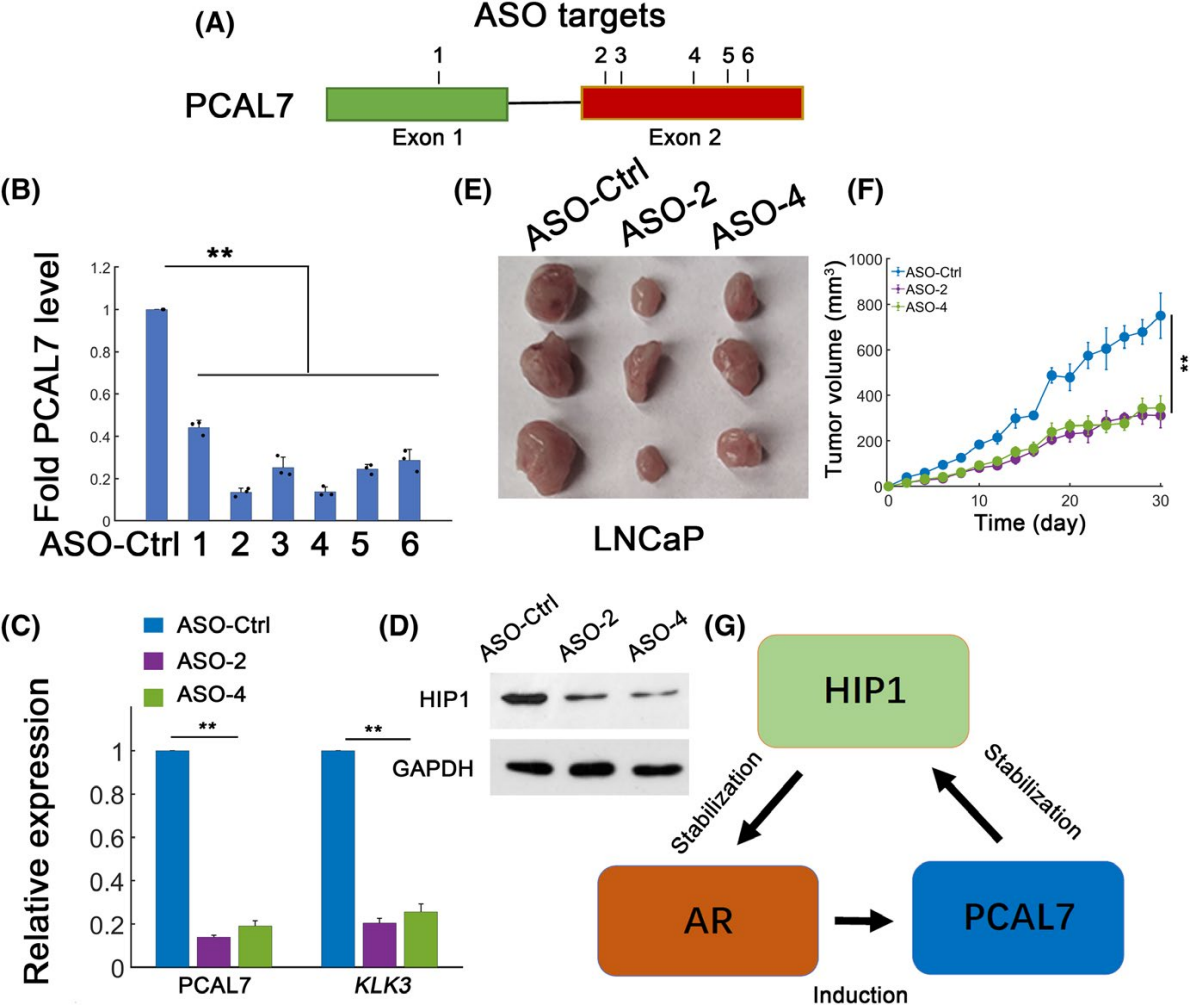
PCAL7 silence by ASOs inhibits prostate cancer. A, Positions of PCAL7 ASO target sites (six oligos). B, LNCaP cells were transfected with six independent ASOs for PCAL7. Efficacies were evaluated by qPCR. C, Effect of ASOs treatment (ASO‐Ctrl, ASO‐2, and ASO‐4) on PCAL7 and AR target gene expression (*KLK3*). D, HIP1 protein expression with ASO treatment in LNCaP cells. E, Effect of ASOs (ASO‐Ctrl, ASO‐2, and ASO‐4) treatment on xenograft tumor growth. F, Measurements of temporal tumor growth. G, A proposed model for PCAL7 in AR signaling. **P < .01

The potential in vivo effects of PCAL7 were further evaluated. We used antisense oligonucleotides (ASOs) to target PCAL7 and deplete intrinsic PCAL7 expression.^14^ Six oligos were designed to target PCAL7 (Figure 5A). Treatment with ASOs dramatically reduced PCAL7 expression (Figure 5B). As observed, ASO‐2 and ASO‐4 most significantly decreased PCAL7 abundance and were chosen for further study (Figure 5B). PCAL7‐ASOs strongly reduced *HIP1* and *KLK3* transcript expression implying accelerated HIP1 turnover and impaired AR signaling (Figure 5C,D). Mice harboring LNCaP xenografts with PCAL7 ASO‐2 or ASO‐4 revealed significant decreases in tumor growth (Figure 5E,F). Importantly, PCAL7 ASOs treatment did not significantly affect mice body weights and biochemical parameters suggesting that ASO treatment was a safe strategy (Figure S2A,B). These data collectively showed that PCAL7 potentiated proliferation of AR‐dependent prostate cancer and may act as a therapeutic target.

## 4 DISCUSSION

Even castration resistance prostate cancer remains dependency on AR signaling; therefore, it is highly important to identify potential targets for therapeutic interventions in prostate cancer. Enzalutamide or abiraterone have shown its effectiveness for prostate cancer patients,^15^, ^16^ and novel targets other than AR itself are strongly required for combinatorial therapy.

By comprehensive profiling, we identified a novel AR‐regulated lncRNA PCAL7. PCAL7 stabilizes HIP1 protein via PCAL7‐HIP1 interaction. Since HIP1 has a defined positive role to activate AR signaling pathway,^13^ the PCAL7‐HIP1 interaction may indirectly augment AR signaling and create a novel positive feedback loop in AR positive or dependent prostate cancer (Figure 5G). Several previous studies on lncRNAs such as SNHG10^17^ and CCAT2^18^ also involve positive feedback loops implying that implementing positive feedback regulation may act as a design principle during cancer progression. Positive feedback has emergent properties leading to bistability and cell fate decision.^19^ The co‐existence of multiple positive feedbacks may provide redundancy and robustness during cancer progression.^20^ Therefore, regulating positive feedback loops through intricate design (eg, antisense oligonucleotides) may provide an effective strategy to inhibit prostate cancer progression.

We found that PCAL7 interacts with HIP1, which suggests that PCAL7 might be an upstream regulator for AR signaling pathway. Therefore, PCAL7 regulation could provide an alternative regime in addition to direct AR targeting. Meanwhile, manipulation of PCAL7 expression might be required in specific patients with resistance to AR‐targeted therapies. Conventional RNA interference has many drawbacks such as inefficient cellular uptake and decreased stability.^6^ Instead, ASOs with phosphorothioate have shown enhanced efficiency to deplete prostate cancer cells.^21^ Meanwhile, in a recent clinical trial, a specific ASO for IONIS‐APO(a)_Rx_ has been used to regulate apolipoprotein expression during calcific aortic valve stenosis (CAVS) treatment.^22^ Interesting, several ASOs were designed in current work which effectively diminish PCAL7 expression (ASO‐2 and ASO‐4, especially). As demonstrated above, these two ASOs can impede in vivo tumor growth without remarkable side effects. These data may support the notion that targeting PCAL7 might be an effective strategy to eradicate prostate cancer cells.

Furthermore, PCAL7 can interact with HIP1 to stabilize HIP1 proteins. Notably, RNAs have shown to interact with target mRNAs through RNA‐RNA interaction.^23^ Whether PCAL7 can interact with other RNA targets remains to be determined. Meanwhile, it is still unclear whether PCAL7‐HIP1 interaction requires additional protein or RNA regulators in vivo.^24^ Whether PCAL7 presents lineage‐specific expression remains elusive and should be explored in future.

Previous work has demonstrated that HIP1 protein is abundantly overexpressed in prostate cancer.^13^ HIP1 can stabilize AR proteins and translocate to the nucleus to facilitate androgen‐mediated transcriptional programs leading to hyperactivated AR signaling.^13^ Stabilization of cellular HIP1 levels therefore plays an essential role for AR activation and AR‐dependent prostate cancer progression. We have shown a novel association between PCAL7 and HIP1 by RNA‐protein interaction. PCAL7‐HIP1 interaction greatly increased HIP1 protein abundance to maintain AR hyperactivation. In conclusion, we showed that PCAL7 is a novel AR‐regulated lncRNA which can decrease HIP1 degradation and create a positive feedback loop in AR signaling. Therefore, PCAL7 can serve as a potential target for therapeutic intervention during prostate cancer treatment.

## Supporting information

App S1

## Data Availability

The data that support the findings of this study are available from the corresponding author upon reasonable request.

## References

[1] Siegel RL , Miller KD , Jemal A . Cancer statistics, 2019. CA Cancer J Clin. 2019;69(1):7‐34. 30620402 10.3322/caac.21551

[2] Shen MM , Abate‐Shen C . Molecular genetics of prostate cancer: new prospects for old challenges. Genes Dev. 2010;24(18):1967‐2000. 20844012 10.1101/gad.1965810 PMC2939361

[3] Kregel S , Wang C , Han X , et al. Androgen receptor degraders overcome common resistance mechanisms developed during prostate cancer treatment. Neoplasia. 2020;22(2):111‐119. 31931431 10.1016/j.neo.2019.12.003 PMC6957805

[4] Lu‐Yao GL , Albertsen PC , Moore DF , et al. Fifteen‐year survival outcomes following primary androgen‐deprivation therapy for localized prostate cancer. JAMA Intern Med. 2014;174(9):1460‐1467. 25023796 10.1001/jamainternmed.2014.3028 PMC5499229

[5] Fujimoto N . Role of the androgen‐androgen receptor axis in the treatment resistance of advanced prostate cancer: from androgen‐dependent to castration resistant and further. J UOEH. 2016;38(2):129‐138. 27302726 10.7888/juoeh.38.129

[6] Adams BD , Parsons C , Walker L , Zhang WC , Slack FJ . Targeting noncoding RNAs in disease. J Clin Invest. 2017;127(3):761‐771. 28248199 10.1172/JCI84424 PMC5330746

[7] Zhang Y , Pitchiaya S , Cieslik M , et al. Analysis of the androgen receptor‐regulated lncRNA landscape identifies a role for ARLNC1 in prostate cancer progression. Nat Genet. 2018;50(6):814‐824. 29808028 10.1038/s41588-018-0120-1 PMC5980762

[8] Lingadahalli S , Jadhao S , Sung YY , et al. Novel lncRNA LINC00844 regulates prostate cancer cell migration and invasion through AR signaling. Mol Cancer Res. 2018;16(12):1865‐1878. 30115758 10.1158/1541-7786.MCR-18-0087

[9] Chakravarty D , Sboner A , Nair SS , et al. The oestrogen receptor alpha‐regulated lncRNA NEAT1 is a critical modulator of prostate cancer. Nat Commun. 2014;5:5383. 25415230 10.1038/ncomms6383 PMC4241506

[10] Lanz RB , McKenna NJ , Onate SA , et al. A steroid receptor coactivator, SRA, functions as an RNA and is present in an SRC‐1 complex. Cell. 1999;97(1):17‐27. 10199399 10.1016/s0092-8674(00)80711-4

[11] Dijkstra S , Leyten GH , Jannink SA , et al. KLK3, PCA3, and TMPRSS2‐ERG expression in the peripheral blood mononuclear cell fraction from castration‐resistant prostate cancer patients and response to docetaxel treatment. Prostate. 2014;74(12):1222‐1230. 25043536 10.1002/pros.22839

[12] Yan X , Zhang D , Wu W , et al. Mesenchymal stem cells promote hepatocarcinogenesis via lncRNA‐MUF interaction with ANXA2 and miR‐34a. Cancer Res. 2017;77(23):6704‐6716. 28947421 10.1158/0008-5472.CAN-17-1915

[13] Mills IG , Gaughan L , Robson C , et al. Huntingtin interacting protein 1 modulates the transcriptional activity of nuclear hormone receptors. J Cell Biol. 2005;170(2):191‐200. 16027218 10.1083/jcb.200503106 PMC2171420

[14] Meng L , Ward AJ , Chun S , Bennett CF , Beaudet AL , Rigo F . Towards a therapy for Angelman syndrome by targeting a long non‐coding RNA. Nature. 2015;518(7539):409‐412. 25470045 10.1038/nature13975 PMC4351819

[15] Scher HI , Fizazi K , Saad F , et al. Increased survival with enzalutamide in prostate cancer after chemotherapy. N Engl J Med. 2012;367(13):1187‐1197. 22894553 10.1056/NEJMoa1207506

[16] Stein MN , Goodin S , Dipaola RS . Abiraterone in prostate cancer: a new angle to an old problem. Clin Cancer Res. 2012;18(7):1848‐1854. 22451619 10.1158/1078-0432.CCR-11-1805 PMC3761800

[17] Lan T , Yuan K , Yan X , et al. LncRNA SNHG10 facilitates hepatocarcinogenesis and metastasis by modulating its homolog SCARNA13 via a positive feedback loop. Cancer Res. 2019;79(13):3220‐3234. 31101763 10.1158/0008-5472.CAN-18-4044

[18] Chen F , Bai G , Li Y , Feng Y , Wang L . A positive feedback loop of long noncoding RNA CCAT2 and FOXM1 promotes hepatocellular carcinoma growth. Am J Cancer Res. 2017;7(7):1423‐1434. 28744394 PMC5523025

[19] Tyson JJ , Chen KC , Novak B . Sniffers, buzzers, toggles and blinkers: dynamics of regulatory and signaling pathways in the cell. Curr Opin Cell Biol. 2003;15(2):221‐231. 12648679 10.1016/s0955-0674(03)00017-6

[20] Kitano H . Biological robustness. Nat Rev Genet. 2004;5(11):826‐837. 15520792 10.1038/nrg1471

[21] Crooke ST , Wang S , Vickers TA , Shen W , Liang XH . Cellular uptake and trafficking of antisense oligonucleotides. Nat Biotechnol. 2017;35(3):230‐237. 28244996 10.1038/nbt.3779

[22] Viney NJ , van Capelleveen JC , Geary RS , et al. Antisense oligonucleotides targeting apolipoprotein(a) in people with raised lipoprotein(a): two randomised, double‐blind, placebo‐controlled, dose‐ranging trials. Lancet. 2016;388(10057):2239‐2253. 27665230 10.1016/S0140-6736(16)31009-1

[23] Engreitz JM , Sirokman K , McDonel P , et al. RNA‐RNA interactions enable specific targeting of noncoding RNAs to nascent Pre‐mRNAs and chromatin sites. Cell. 2014;159(1):188‐199. 25259926 10.1016/j.cell.2014.08.018 PMC4177037

[24] Lebedeva S , Jens M , Theil K , et al. Transcriptome‐wide analysis of regulatory interactions of the RNA‐binding protein HuR. Mol Cell. 2011;43(3):340‐352. 21723171 10.1016/j.molcel.2011.06.008

