# Supplementary material for: The long non‐coding RNA PCAL7 promotes prostate cancer by strengthening androgen receptor signaling
Source: J Clin Lab Anal. 2020 Nov 21;35(2):e23645. doi: 10.1002/jcla.23645 (PMC7891507; doi:10.1002/jcla.23645)
Supplement: Supplementary file 1 — App S1 [file JCLA-35-e23645-s001.docx]

**Supplemental methods**

**RNA-sequencing (RNA-seq)**

The total RNAs were extracted from VCaP or LNCaP cells after DHT treatment with miRNeasy kit (Qiagen) and the quality was evaluated by Agilent Bioanalyzer. The samples were subject to Illumina HiSeq 3000 and performed in BGI (Shenzhen, China). The library passing quality control was conducted by removing adaptors during sequencing followed by alignment to reference genome, GRCh38. Expression was quantified in FeatureCounts using MiTranscriptome (1). The relative expression (FPKM) was normalized usig edgeR (2). The resulting *P* values were adjusted using the Benjamini and Hochberg’s approach for controlling the false discovery rate. Genes with a two-sided *P* value < 0.05 and fold change > 2.0 were regarded as statistically significant genes.

**RNA extraction and cDNA synthesis**

Total RNAs from cells were extracted by the miRNeasy kit (Qiagen) according to the manufacturer’s guidelines. Superscript III and random primers (Invitrogen) were used for cDNA synthesis.

**RNA pulldown assay and mass spectrometry (MS)**

Biotin-labeled RNAs were transcribed with Biotin RNA Labeling Mix and T7 RNA polymerase (Roche) followed by purification with the RNeasy Mini Kit (Qiagen). Total RNAs were then annealed, mixed with extracts from RIP buffer and incubated for 3h at 20℃. Streptavidin agarose beads were incubated at 20℃ for 2h. Beads were extracted by Trizol reagent for quantitative PCR (*q*PCR). Electrophoresis was used to separate specific bands for further mass spectrometry (MS). Samples from mass spectrometry was sent to and analyzed at BGI at Beijing, China.

**Chromatin immunoprecipitation (ChIP)-qPCR**

Briefly, the enrichment analysis of AR in PCAL7 promoter region was conducted with the EZ-ChIP Kit (Thermo, USA) using AR and IgG antibodies. Then, qPCR was used to analyze enriched DNA. 5×10^6^ cells were transfected as indicated. 1 h after DHT treatment, cells were crossed with formaldehyde. Then, DNA extracts from lysed cells were sonicated using Biorupter (Diagenode) and subject to immunoprecipitation with AR antibody overnight in conjugation with protein G magnetic beads (Sigma). The washed and reverse crosslinked beads with 5% input was subject to DNA isolation using PCR-purification kit (Qaigen). The enrichment was assessed by PCR. The primers were designed and purchased from Invitrogen.

**Plasmids and siRNAs**

The full-length PCAL7 was obtained from Integrated DNA Technologies (IDT) and inserted into pcDNA3.1 vector at *ApaI* and *EcoRI* sites. The transfection was conducted using Lipofectamine 3000 (Invitrogen). Insert sequence was further validated by Sanger sequencing. VCaP and LNCaP cells were first cultured for 24 hours and then transfected with 3 nM siRNAs obtained from IDT using Lipofectamine RNAiMAX transfection reagent (Invitrogen) following the user guideline. 36 hours later, transfected was performed again similarly using 3 nM siRNAs. Then, cells treated with either ethanol Vehicle control (Veh) or 10 nM DHT were analyzed by RT-qPCR, western or *in vitro* assays. The non-targeting control siRNA (si-NC) was used as the control. Primers were listed in Table S1.

**Immunohistochemistry**

Paraffin-embedded sections (5 μm) were deparaffinized, washed in xylene. Slides were heated with SSC buffer at 100℃ and then cooled down. The blockage was done by 3% H_2_O_2_ for 20 min. Citrate buffer (pH 6.5, 20 mmol/L) was added to retrieve antigens. Cooldown was performed by TBST wash twice. After treatment with blocking solution at 4℃ for 30 min, primary antibodies were covered for 3 h and then washed by TBST twice. BrdU Staining Kit for Flow Cytometry eFluor™ 450 (Invitrogen) and anti-cleaved caspase-3 antibody (AB3623, Sigma) were used. Positive staining was evaluated with a Leica DM IRE2 microscopy (Leica).

**Migration assay**

1×10^5^ LNCaP and VCaP cells transfected with si-NC, si-PCAL7, empty pcDNA or pcDNA-PCAL7 were located in Boyden’s Transwell Chamber (CST) in DMEM with 0.5% FBS. The DMEM with 10% FBS containing either Veh or DHT was loaded at bottom. After an incubation for 36 hours, cells moving into the bottom chambers were fixed with 4% paraformaldehyde (No.16005, Sigma) and subject to staining by 0.4% crystal violet (No.C0775, Sigma) and the results were visualized by an inverted bright field microscopy.

**Antisense oligonucleotides (ASOs)**

The ASOs were designed and purchased from IDT. 50 nM ASOs were used for transfection using Lipofectamine RNAiMAX (Invitrogen). Transfection lasted for 36 hours. The antisense oligos were listed in Table S1.

**Fluorescence *in situ* hybridization (FISH)**

Fluorescence conjugated probes were designed by and obtained from IDT. The non-denaturing conditions were applied to treat samples. Then, fluorescence-conjugated probes were appended. Samples were counterstained with DAPI and visualized by a confocal microscopy.

**Cell proliferation**

The proliferation of cells was performed in 12-well plates. Cell attachment was allowed. Proliferation data were recorded by IncuCyte live-cell imaging assay system (Essen Biosciences) according to the manufacturer’s instructions.

**Western blot**

Cells were lysed in RIPA lysis buffer (V900854, Sigma) with protease inhibitor cocktail (P8340, Sigma). Protein concentrations were determined by DC protein assay (BioRad) and lysates were boiled. The protein extracts were subject to SDS-PAGE separation with polyvinylidene difluoride membrane. The membranes were blocked in blocking buffer (4% milk in 0.2% TBST) and incubated overnight in a refrigerator with specific primary antibodies. After being washed by TBST for three times, membranes were coated with HRP-conjugated secondary antibody. Signals were quantified using ImageJ.

**RNA immunoprecipitation (RIP)**

The MS2bp-MS2bs-based RIP assay was performed as previously described (3). In brief, the pcDNA3.1-Flag-MS2bp and pcDNA3.1-PCAL7-MS2bs were co-transfected into LNCaP cells. After 36 hours, cells were harvested and then lysed by mild lysis buffer together with RNase inhibitor. Immunoprecipitation was performed with anti-FLAG magnetic beads (Invitrogen). Complexes of RNA and its partners were extracted by Trizol (Invitrogen).

***In vivo* experiments**

Athymic nude mice (4~5-week-old, male) were inoculated subcutaneously with LNCaP cells suspended in a Matrigel scaffold in posterior dorsal flank (6×10^6^ cells/site, totally two sites). After the tumor size approximated 100 mm^3^, mice were randomized into three groups, each group was treated with ASO-2, ASO-4 or control ASO (ASO-Ctrl) at a dose of 70 mg/kg. ASOs were subcutaneously injected into the scapulae every two days for a total of 30 days. Tumor size was calculated by a caliper. 1/3 solid tumors were placed in 10% formalin buffer, whereas the remaining ones were snap-frozen and stored in a -80℃ refrigerator. The experimental protocols were approved by the Institutional Animal Care and Use Committee at the Seventh Medical Center of PLA General Hospital.

**Luciferase reporter assay**

The AR reporter assay was performed using the Dual Luciferase Reporter Gene Aassy Kit (qcBio) following the manufacturer’s protocols. Briefly, cells transfected with siRNAs and reporter vectors using Lipofectamine 2000 (Thermo Fisher Scientific). 36 hours later, DHT or Veh treated control was added to activate AR signaling. The luciferase assay was performed 6 hours after DHT stimulation. Normalization was done with reference to the ratio of firefly/Renilla activity and cell numbers.


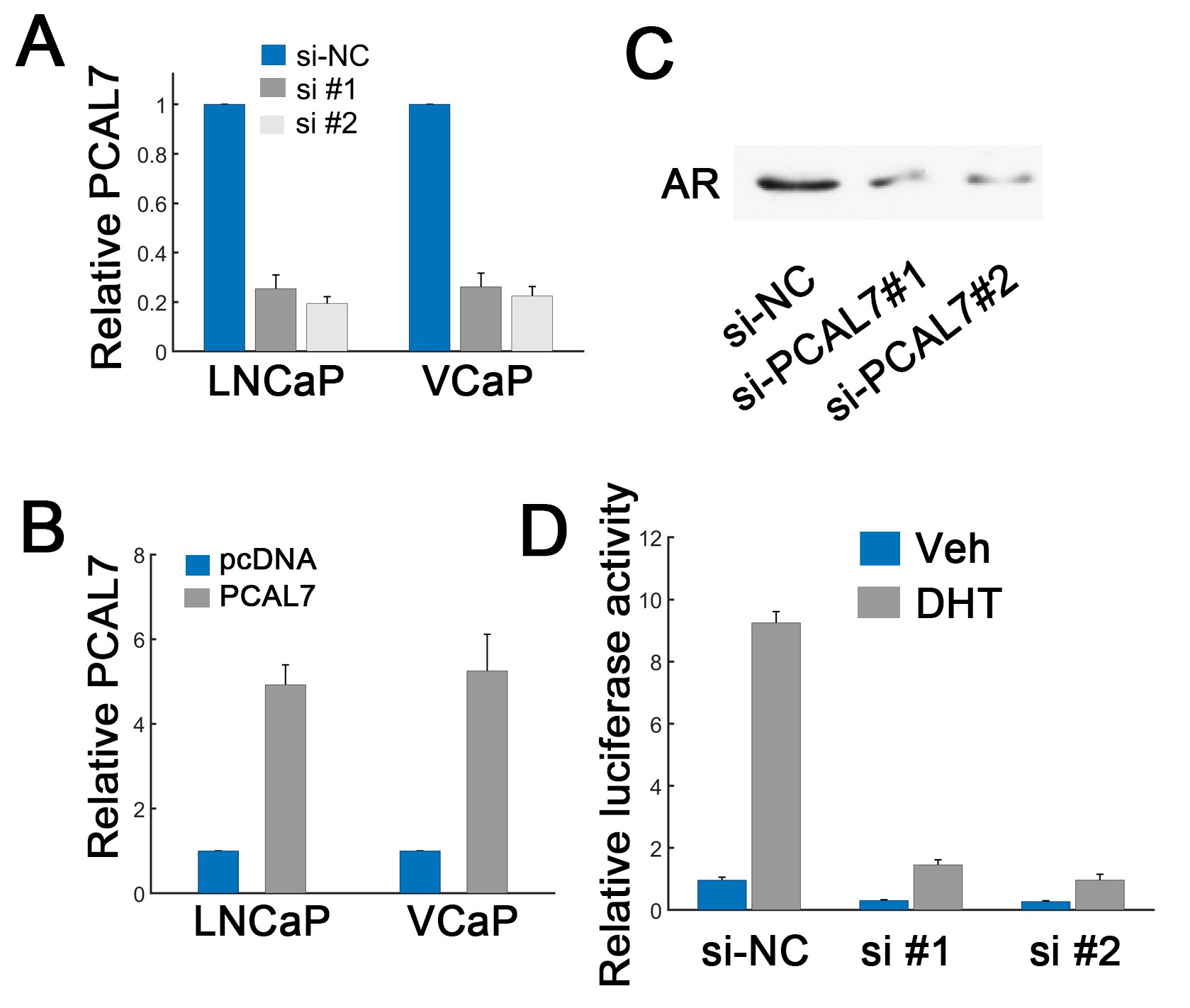


Figure S1. PCAL7 silence or overexpression efficiency on AR activity. (A) The efficiency of PCAL7 knockdown in LNCaP and VCaP cells. Two siRNAs (si-PCAL7-1 and si-PCAL7-2) were used. si-PCAL7#2 was used in main text for higher efficiency. (B) Effect of PCAL7 overexpression. (C) PCAL7 silence on AR protein expression. The siRNAs for negative control (si-NC), PCAL7-1, PCAL7-2 were used. (D) Effect of PCAL7 knockdown on AR reporter gene activity.


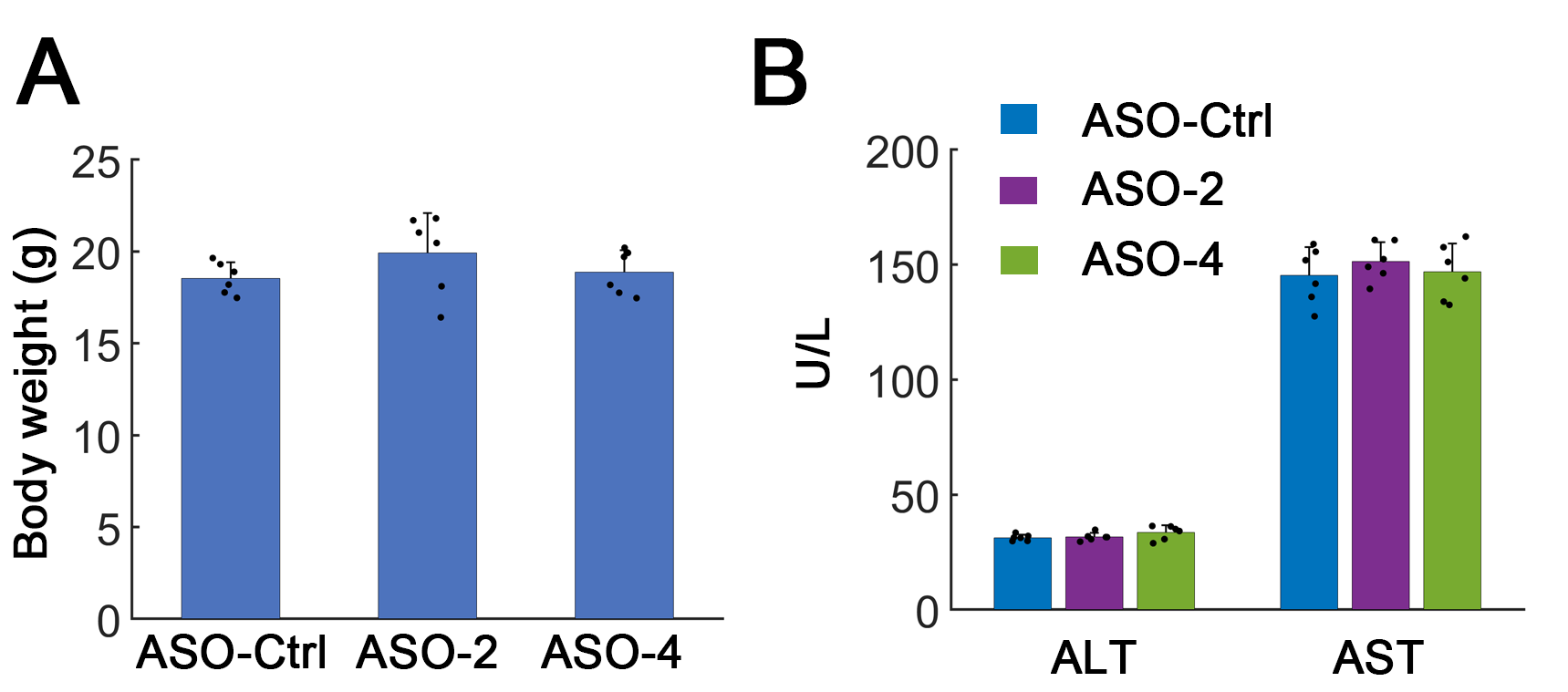


Figure S2. Effect of ASO treatment. (A) Body weights after treatment with ASO-Ctrl, ASO-2 or ASO-4. (B) Seral AST or ALT levels after ASO-Ctrl, ASO-2 or ASO-4 treatment (ALT: alanine aminotransferase; AST: aspartate aminotransferase).

**Table S1.** Primers and antibodies

| **Name** | **Sequences** |
| --- | --- |
| PCAL7-F | GACCCAATACTGTCGCCCCACACTAT |
| PCAL7-R | TAATCAATATCCAACGGCAGCAT |
| Antisense-F | CCACTTGCAGAAGTACA |
| Antisense-R | GCATTACAATTTCGTTGGAACAAACAC |
| *GAPDH*-F | ACAGTGCCCTCAGGAGGATTC |
| *GAPDH*-R | AGAGACGGGAGGTGAC |
| *U6*-F | CATACTGGCTCAATCAACAATAAGAA |
| *U6*-R | TAACACAGACGCAAGGCTTTGT |
| *HIP1*-F | TTATGATTGCATCAAACTTTGTC |
| *HIP1*-R | CCCTTCGCCTTCGATGTCAGTT |
| PCAL7 1-90-F  1-90-R | GGTTGACTAGCTATTGCATGAAGG  AAGGAGTTGGAGCATGAAC |
| 1-286-F | TAGGAGGCATAAATATCTCACGTTAT |
| 1-286-R | GCGAGCGGAGTAAAGGCGGTGTGA |
| 287-345-F | AGTAAGATACGGAGCTCACGAT |
| 287-345-R | GCAATCTCAGGGTTTATACTGTG |
| 346-648-F | AGTAAGATACGGGTCCAGCGAT |
| 346-648-R | TTTGCCAGGTCCGATTTAACGT |
| siPCAL7-1 | CGCTTTCCAGCCTGTGC |
| siPCAL7-2 | GCATGTAAGTCCTATT |
| *si-AR*1 | AAGCGTCTCTTCCATG |
| *si-AR*2 | TTATAGGGAATGAGGT |
| *si-NC* | TACGGCGAGTGCTTAT |
| *KLK3*-F | GGGTGCTTGCAGGGCACAAG |
| *KLK3*-R | GGTGGTACGCTGGACAGTCACT |
| PCAL7-promoter-F | TAGATTAATAACCCCAACAT |
| PCAL7-promoter-R | GCTCTGGTCAAGCTGAATTTGCT |
| *AR*-F | ATGGGCTGAACTTGGTGAGCTGTG |
| *AR*-R | GGAGCAGTGGGTAGCTTA |
| ASO-1 | mUmCmUmVmGATCGGCGATTmGmTmGmGmU |
| ASO-2 | mUmAmGmUmCCCTCTGAACmAmAmUmCmU |
| ASO-3 | mGmAmUmGmGCTATGCATAmGmGmAmAmU |
| ASO-4 | mUmUmAmCATTGTGGCAGTmAmCmCmUmG |
| ASO-5 | mAmUmCmGATAGCGGTACAmUmGmAmUmG |
| ASO-6 | mGmAmUmAmCTGTGATCGmUmGmUmGmU |
| ASO-Control | mUmCmAmUmGGGCTGAATTmCmAmUmGmC |
| **Antibodies or reagents (Catalog NO.)** | **Sources** |
| AR (SAB4501575) | Sigma |
| GAPDH (G8795) | Sigma |
| HIP1 (SAB4200391) | Sigma |

**Table S2:** Clinicopathological features.

|  |  | PCAL7 expression | |
| --- | --- | --- | --- |
| Features | Case No. | Low/High | *P* value |
| **Age** |  |  |  |
| < 60 | 50 | 27/23 | 0.278 |
| ≥ 60 | 54 | 25/29 |  |
| **TNM stage** |  |  |  |
| I/II | 47 | 34/13 | <0.001 |
| III/IV | 57 | 18/39 |  |
| **Tumor size** |  |  |  |
| < 2.5 cm | 45 | 30/15 | 0.003 |
| ≥ 2.5 cm | 59 | 22/37 |  |
| **Metastasis** |  |  |  |
| Absent | 48 | 31/17 | 0.005 |
| Present | 56 | 21/35 |  |

TNM: tumor (T), the extent of spread to the lymph nodes (N), and the presence of metastasis (M) (* P < 0.05, ** *P* <0.01). The median value was used as the cut-off.

**Table S3:** Putative binding factors by mass spectrometry

| Accession | Name | Species | | Genes | Scores |
| --- | --- | --- | --- | --- | --- |
| O00291 | Huntingtin-interacting protein 1 | Homo sapiens | *HIP1* | | 1920.68 |
| P18887 | DNA repair protein XRCC1 | Homo sapiens | *XRCC1* | | 1567.53 |
| Q5SSJ5 | Heterochromatin protein 1-binding protein 3 | Homo sapiens | *HP1B3* | | 1182.22 |
| P31749 | RAC-alpha serine/threonine-protein kinase 1 | Homo sapiens | *Akt1* | | 919.14 |
| Q13315 | Serine/threonine-protein kinase ATM | Homo sapiens | *ATM* | | 1028.78 |
| Q8NHM5 | Lysine-specific demethylase 2B | Homo sapiens | *KDM2B* | | 1240.23 |

Please refer to UniProt for details, <http://www.uniprot.org/>

**Reference**

1. Iyer MK, Niknafs YS, Malik R, Singhal U, Sahu A, Hosono Y, et al. The landscape of long noncoding RNAs in the human transcriptome. Nat Genet. 2015;47(3):199-208.

2. Robinson MD, McCarthy DJ, Smyth GK. edgeR: a Bioconductor package for differential expression analysis of digital gene expression data. Bioinformatics. 2010;26(1):139-40.

3. Yan X, Zhang D, Wu W, Wu S, Qian J, Hao Y, et al. Mesenchymal Stem Cells Promote Hepatocarcinogenesis via lncRNA-MUF Interaction with ANXA2 and miR-34a. Cancer Res. 2017;77(23):6704-16.
